# Correction: Incidence of cerebrovascular disease in Peru from 2015 to 2023

**DOI:** 10.1371/journal.pgph.0006062

**Published:** 2026-02-23

**Authors:** Diego Maximiliano Guevara Rodríguez, Juan Diego Pichihua Grandez, Fabrizio Valdivia Dianderas, José Del Carmen Sara

The Data Availability statement for this article is incorrect. The correct statement is: The raw data are provided in S1 Table.
