# Supplementary material for: Incidence of cerebrovascular disease in Peru from 2015 to 2023
Source: PLOS Glob Public Health. 2025 May 29;5(5):e0004559. doi: 10.1371/journal.pgph.0004559 (PMC12121790; doi:10.1371/journal.pgph.0004559)
Supplement: S2 Table — (DOCX) [file pgph.0004559.s002.docx]

**S2 Table. Frequency of hospital discharges due to cerebrovascular disease by department**

| **Variable** | **Hospital Discharges** | |
| --- | --- | --- |
|  | **N** | **%** |
| **Departments** |  |  |
| AMAZONAS | 1148 | 1.28 |
| ANCASH | 1130 | 1.26 |
| APURIMAC | 558 | 0.62 |
| AREQUIPA | 3867 | 4.31 |
| AYACUCHO | 1352 | 1.51 |
| CAJAMARCA | 2670 | 2.97 |
| CALLAO | 5818 | 6.48 |
| CUSCO | 3488 | 3.89 |
| HUANCAVELICA | 194 | 0.22 |
| HUANUCO | 638 | 0.71 |
| ICA | 2483 | 2.77 |
| JUNIN | 3356 | 3.74 |
| LA LIBERTAD | 7496 | 8.35 |
| LAMBAYEQUE | 3549 | 3.95 |
| LIMA | 41205 | 45.90 |
| LORETO | 2028 | 2.26 |
| MADRE DE DIOS | 182 | 0.20 |
| MOQUEGUA | 412 | 0.46 |
| PASCO | 548 | 0.61 |
| PIURA | 2807 | 3.13 |
| PUNO | 1574 | 1.75 |
| SAN MARTIN | 1754 | 1.95 |
| TACNA | 532 | 0.59 |
| TUMBES | 210 | 0.23 |
| UCAYALI | 777 | 0.87 |
