# Supplementary material for: Incidence of cerebrovascular disease in Peru from 2015 to 2023
Source: PLOS Glob Public Health. 2025 May 29;5(5):e0004559. doi: 10.1371/journal.pgph.0004559 (PMC12121790; doi:10.1371/journal.pgph.0004559)
Supplement: S3 Table — (DOCX) [file pgph.0004559.s003.docx]

**S3 Table. Specified diagnosis frequency by department**

| **Variable** | **Total (n)** | **Specified Diagnosis (%)** |
| --- | --- | --- |
| **Departments** |  |  |
| AMAZONAS | 1148 | 4.09 |
| ANCASH | 1130 | 29.82 |
| APURIMAC | 558 | 43.01 |
| AREQUIPA | 3867 | 45.44 |
| AYACUCHO | 1352 | 30.92 |
| CAJAMARCA | 2670 | 22.06 |
| CALLAO | 5818 | 31.45 |
| CUSCO | 3488 | 31.91 |
| HUANCAVELICA | 194 | 47.42 |
| HUANUCO | 638 | 37.15 |
| ICA | 2483 | 21.10 |
| JUNIN | 3356 | 31.88 |
| LA LIBERTAD | 7496 | 44.56 |
| LAMBAYEQUE | 3549 | 33.62 |
| LIMA | 41205 | 35.43 |
| LORETO | 2028 | 45.86 |
| MADRE DE DIOS | 182 | 23.63 |
| MOQUEGUA | 412 | 15.53 |
| PASCO | 548 | 30.84 |
| PIURA | 2807 | 30.53 |
| PUNO | 1574 | 42.44 |
| SAN MARTIN | 1754 | 23.15 |
| TACNA | 532 | 32.71 |
| TUMBES | 210 | 30.00 |
| UCAYALI | 777 | 22.14 |
