# Supplementary material for: Incidence of cerebrovascular disease in Peru from 2015 to 2023
Source: PLOS Glob Public Health. 2025 May 29;5(5):e0004559. doi: 10.1371/journal.pgph.0004559 (PMC12121790; doi:10.1371/journal.pgph.0004559)
Supplement: Attachment — Submitted filename: Response to Reviewers.docx [file pgph.0004559.s005.docx]

**Response to Reviewers**

Dear Editor and Reviewers:

This is to acknowledge the response to our submission and the comments made to the publication proposal. We will now proceed to respond each comment:

**Response to Editor:**

1. **The abbreviation CVD is ambiguous, as it can be understood as cardiovascular disease**

We thank you for your comment. We proceeded to abbreviate cerebrovascular disease as “stroke” because there is literature that abbreviates cerebrovascular disease in that way considering ischemic as well as hemorrhagic cerebrovascular disease.

2. **The categories of health facilities are confusing, and information is insufficient**

Thanks for the comment. In the study design section, we have added more information from line 184 to 222 regarding the categories of health facilities.

3. **The method for calculating the incidence rate is not clear and the authors might consider providing an equation to simplify the use of Excel.**

We appreciate the observation; in the data analysis section we have provided more detail for the estimation of the cumulative incidence (Proportional Incidence). We specify the numerator, denominator and constant (k = 10,000).

The text is presented now in the following way (line 260 to 263):

To estimate incidence, we used hospital admissions for CVD in the numerator, and -as population at risk, the denominator- we used population projections by years for sex, age and departments from INEI [19,20]. The cumulative incidence was expressed per 10,000 inhabitants.

4. **Is there any reason to use the word per 10,000 “inhabitants”?**

Thank you for your observation. For the estimation of cumulative incidence, we make use of a constant (K), which we decided to define as 10,000. This presentation allows for a more intuitive interpretation of the population at risk, i.e., 3.11 cases per 10,000 people in the population at risk. Traditionally, the wording is presented as 3.11 cases per 10,000 persons.

**Response to Reviewer 1:**

1. **The abbreviation CVD should be avoided because of its ambiguity.**

We thank you for your comment. We proceeded to abbreviate cerebrovascular disease as “stroke” because there is literature that abbreviates cerebrovascular disease in that way considering ischemic as well as hemorrhagic cerebrovascular disease.

2. **The section discussing CVD as the “second leading cause of cardiovascular mortality” should be revised to clarify that the term refers to cerebrovascular disease. A clearer distinction between cardiovascular and cerebrovascular disease will reduce any ambiguity for readers.**

We agree and appreciate the observation. To avoid this ambiguity, we proceeded to change the abbreviation of cerebrovascular disease to stroke. In addition, the Global Burden Disease 2021 considers cerebrovascular disease within cardiovascular conditions, hence the statement “second leading cause of cardiovascular mortality”.

3. **More rigorous statistical methods, such as regression analysis, to account for potential confounders (e.g., socioeconomic status and lifestyle factors) would add depth to the analysis and strengthen the robustness of the findings.**

We thank you for your comment and suggestion. However, our main objective is to describe the incidence of cerebrovascular disease considering age group, sex and regions of Peru. We present a description of the distribution of this condition according to these variables. We consider that our study is a starting point for an analysis that explores the determinants of CVD in the Peruvian population. Furthermore, the availability of variables of interest in the database analyzed limits the possibility of the analysis suggested by the reviewer.

4. **A more detailed analysis of limitations related to underreporting, coding errors, and missing data would improve transparency and help contextualize the results of the study.**

Agreed. We have added information from line 473 to 498 about underreporting and coding errors and how this same limitation is mitigated using the SUSALUD database.

5. **The conclusions could be strengthened with more specific public health recommendations, particularly for improving the diagnosis and treatment of cerebrovascular disease in regions with higher incidence rates.**

Agreed. We appreciate the comment, we have expanded the conclusion (from line 527 to 542) with more specific recommendations.

**Response to Reviewer 2:**

1. **The rationale as well as implication of the study is not explicitly stated in the Introduction section; such as, more explanation needed regarding the relationship between cerebrovascular disease (CVD) incidence and health resource planning. It is not also obvious from the introductory lines about how the real incidence of CVD will help mitigating the inequalities in accessing healthcare among the population of Peru.**

Agreed. We appreciate the comment, we have added information from line 74 to 77 and from line 128 to 131 to allow a better understanding of the rationale and implications of the study.

2. **The study, although acknowledged the limitation of not including non-hospitalization records such as outpatient cases and mild episodes; but did not elicit the rationale behind this limitation and how this challenge was minimized as those cases cannot be left behind to calculate the real incidence of CVD.**

We appreciate the observation raised. Indeed, the clinical presentation of cerebrovascular disease (CVD) is generally severe, requiring specialized care in an appropriate health center. We recognize that there is a possibility that some cases of CVD may not be hospitalized, either because patients do not arrive alive at the hospital or because they are not transferred to a nearby health center. Because of this, these cases are not found in the hospital discharge records, which limits the inclusion of all possible CVD events in our database. However, these cases do not generate burden at the hospital level. Furthermore, with this study we aim to estimate the incidence of cases that merited treatment at the hospital level for future planning of health services.

Finally, we take this observation into account and will incorporate it in the limitations section in a clear and summarized way from line 493 to 496, to provide a more precise description of the study's constraints.

3. **The Conclusion section seems like a repetition of the findings. Also, the concluding remark such as, the relationship of study findings with increased hospital beds is not properly explained throughout the paper.**

Agreed. We appreciate the comment, we have expanded the conclusion section from line 527 to 542 for a better understanding.

We are grateful for the suggestions made, and hope that our responses have helped to clarify and improve the quality of our manuscript.

Kind regards,

Diego Maximiliano Guevara Rodriguez, Juan Diego Pichihua Grandez, Fabrizio Rafael Valdivia Dianderas and Jose Carlos del Carmen Sara
